# Supplementary figures and images for: Evaluation of transabdominal and transperineal ultrasound-derived prostate specific antigen (PSA) density and clinical utility compared to MRI prostate volumes: A feasibility study
Source: PLoS One. 2022 Sep 9;17(9):e0274014. doi: 10.1371/journal.pone.0274014 (PMC9462719; doi:10.1371/journal.pone.0274014)

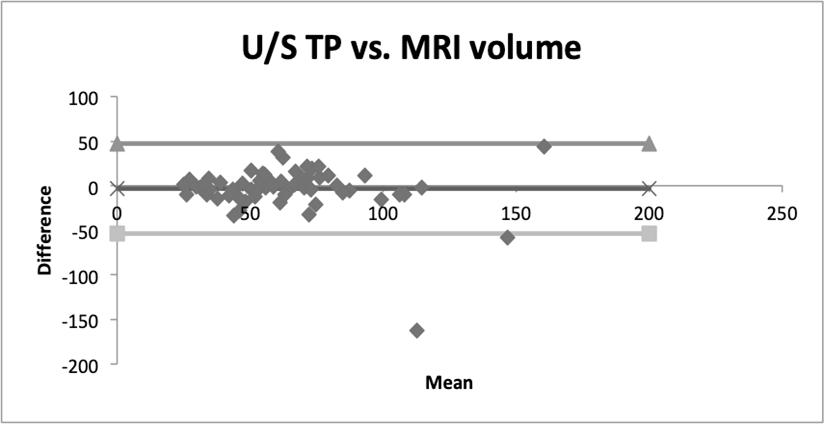

Supplement: S1 Fig — The middle solid line represents the mean agreement difference between MRI and US TP volumes, the lower line represents the lower limit of agreement between the two techniques ([mean– 1.96 standard deviation (SD) and the upper line represents the upper limit of agreement ([mean + 1.96 SD)] between the two techniques. (TIF) [file pone.0274014.s001.tif]

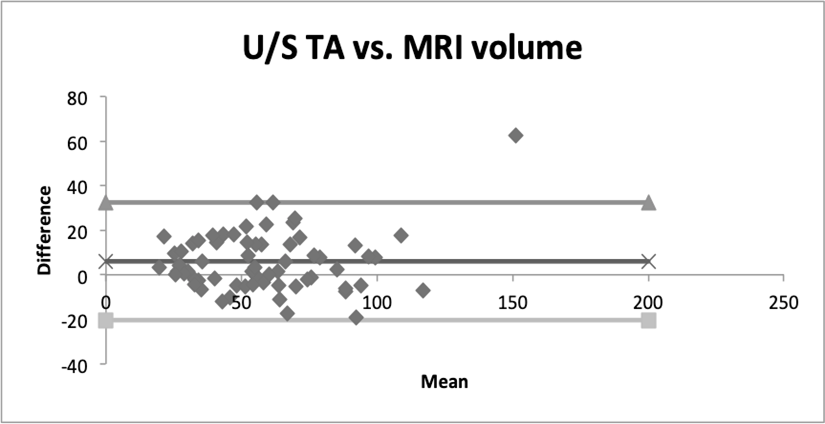

Supplement: S2 Fig — The middle solid line represents the mean agreement difference between MRI and US TP volumes, the lower line represents the lower limit of agreement between the two techniques ([mean– 1.96 standard deviation (SD) and the upper line represents the upper limit of agreement ([mean + 1.96 SD)] between the two techniques. (TIF) [file pone.0274014.s002.tif]
