# Supplementary material for: Evaluation of transabdominal and transperineal ultrasound-derived prostate specific antigen (PSA) density and clinical utility compared to MRI prostate volumes: A feasibility study
Source: PLoS One. 2022 Sep 9;17(9):e0274014. doi: 10.1371/journal.pone.0274014 (PMC9462719; doi:10.1371/journal.pone.0274014)
Supplement: Attachment — Submitted filename: Response to Reviewers Comments.docx [file pone.0274014.s003.docx]

1. Please ensure that your manuscript meets PLOS ONE's style requirements, including those for file naming. The PLOS ONE style templates can be found at

<https://journals.plos.org/plosone/s/file?id=wjVg/PLOSOne_formatting_sample_main_body.pdf> and

<https://journals.plos.org/plosone/s/file?id=ba62/PLOSOne_formatting_sample_title_authors_affiliations.pdf>

2. Thank you for stating the following in the Acknowledgments Section of your manuscript:

“This research was supported by the National Institute of Health Research Cambridge Biomedical Research Centre (BRC-1215-20014). The views expressed are those of the author(s) and not necessarily those of the NIHR or the Department of Health and Social Care. The authors also acknowledge support from Cancer Research UK (Cambridge Imaging Centre grant number C197/A16465), the Engineering and Physical Sciences Research Council Imaging Centre in Cambridge and Manchester and the Cambridge Experimental Cancer Medicine Centre.”

We note that you have provided additional information within the Acknowledgements Section that is not currently declared in your Funding Statement. Please note that funding information should not appear in the Acknowledgments section or other areas of your manuscript. We will only publish funding information present in the Funding Statement section of the online submission form.
**We have removed this from the Acknowledgements section. The funding section included all relevant funding and has been left unchanged.**

Please remove any funding-related text from the manuscript and let us know how you would like to update your Funding Statement. Currently, your Funding Statement reads as follows:
**We have removed tis text from the manuscript.**

“This research was supported by the National Institute of Health Research Cambridge Biomedical Research Centre (BRC-1215-20014). The views expressed are those of the author(s) and not necessarily those of the NIHR or the Department of Health and Social Care. The authors also acknowledge support from Cancer Research UK (Cambridge Imaging Centre grant number C197/A16465), the Engineering and Physical Sciences Research Council Imaging Centre in Cambridge and Manchester and the Cambridge Experimental Cancer Medicine Centre.

The funders had no role in study design, data collection and analysis, decision to publish, or preparation of the manuscript.”
**Please see above. No change required.**

Please include your amended statements within your cover letter; we will change the online submission form on your behalf.

3. In your Data Availability statement, you have not specified where the minimal data set underlying the results described in your manuscript can be found. PLOS defines a study's minimal data set as the underlying data used to reach the conclusions drawn in the manuscript and any additional data required to replicate the reported study findings in their entirety. All PLOS journals require that the minimal data set be made fully available. For more information about our data policy, please see <http://journals.plos.org/plosone/s/data-availability>.
**We have uploaded the dataset file on** [**https://data.mendeley.com/**](https://data.mendeley.com/)**, with the appropriate original data file relevant to this study (DOI:10.17632/2bvdcspxx2.1); it can be accessed via** <https://data.mendeley.com/datasets/2bvdcspxx2/1>

Upon re-submitting your revised manuscript, please upload your study’s minimal underlying data set as either Supporting Information files or to a stable, public repository and include the relevant URLs, DOIs, or accession numbers within your revised cover letter. For a list of acceptable repositories, please see <http://journals.plos.org/plosone/s/data-availability#loc-recommended-repositories>. Any potentially identifying patient information must be fully anonymized.
**We have uploaded the dataset file on** [**https://data.mendeley.com/**](https://data.mendeley.com/)**, with the appropriate original data file relevant to this study.**

Important: If there are ethical or legal restrictions to sharing your data publicly, please explain these restrictions in detail. Please see our guidelines for more information on what we consider unacceptable restrictions to publicly sharing data: <http://journals.plos.org/plosone/s/data-availability#loc-unacceptable-data-access-restrictions>. Note that it is not acceptable for the authors to be the sole named individuals responsible for ensuring data access.
**Not applicable.**

We will update your Data Availability statement to reflect the information you provide in your cover letter.

4. We note you have included a table to which you do not refer in the text of your manuscript. Please ensure that you refer to Table 6 and 7 in your text; if accepted, production will need this reference to link the reader to the Table.
**The reference to the table had been combined within the text as “ (Tables 6, 7)** **”. For added clarity we have changed this to:
*“PSAD at thresholds of >0.15 ng/mL^2^ (Table 6) and > 0.12 ng/mL^2^ (Table 7), respectively.”***

5. Please include your tables as part of your main manuscript and remove the individual files. Please note that supplementary tables (should remain/ be uploaded) as separate "supporting information" files.
**The change has been made.**

6. Please review your reference list to ensure that it is complete and correct. If you have cited papers that have been retracted, please include the rationale for doing so in the manuscript text, or remove these references and replace them with relevant current references. Any changes to the reference list should be mentioned in the rebuttal letter that accompanies your revised manuscript. If you need to cite a retracted article, indicate the article’s retracted status in the References list and also include a citation and full reference for the retraction notice.
**No changes have been made, other than in response to the reviewer’s comments – the relevant changes to the reference are apparent on the tracked version of the resubmitted document.**

[Note: HTML markup is below. Please do not edit.]

Reviewers' comments:

Reviewer's Responses to Questions

**Comments to the Author**

1. Is the manuscript technically sound, and do the data support the conclusions?

The manuscript must describe a technically sound piece of scientific research with data that supports the conclusions. Experiments must have been conducted rigorously, with appropriate controls, replication, and sample sizes. The conclusions must be drawn appropriately based on the data presented.

Reviewer #1: Yes

2. Has the statistical analysis been performed appropriately and rigorously?

Reviewer #1: Yes

3. Have the authors made all data underlying the findings in their manuscript fully available?

The [PLOS Data policy](http://www.plosone.org/static/policies.action#sharing) requires authors to make all data underlying the findings described in their manuscript fully available without restriction, with rare exception (please refer to the Data Availability Statement in the manuscript PDF file). The data should be provided as part of the manuscript or its supporting information, or deposited to a public repository. For example, in addition to summary statistics, the data points behind means, medians and variance measures should be available. If there are restrictions on publicly sharing data—e.g. participant privacy or use of data from a third party—those must be specified.

Reviewer #1: No
**We have uploaded the dataset file on** [**https://data.mendeley.com/**](https://data.mendeley.com/)**, with the appropriate original data file relevant to this study.**

4. Is the manuscript presented in an intelligible fashion and written in standard English?

PLOS ONE does not copyedit accepted manuscripts, so the language in submitted articles must be clear, correct, and unambiguous. Any typographical or grammatical errors should be corrected at revision, so please note any specific errors here.

Reviewer #1: Yes

5. Review Comments to the Author

Please use the space provided to explain your answers to the questions above. You may also include additional comments for the author, including concerns about dual publication, research ethics, or publication ethics. (Please upload your review as an attachment if it exceeds 20,000 characters)

Reviewer #1: In the present study, the authors evaluate the accuracy of surface-based ultrasound-derived PSAD metrics compared to MRI. The patients were prospectively recruited, but no prospective clinical decision making was performed based on the novel data. The authors used MRI-ellipsoid-derived measurements rather than the volumetric segmentation in order to better reflect the clinical routine. The study is well written and a pleasure to read. The results are presented in a concise fashion. For a first small pilot study, it is methodologically sound. I only have a few minor points which should be addressed prior to publication:

1. In accordance with PLOS ONE policy, the authors should make their raw data available with this study. At least, this should be a CSV file containing all the measurements and volumes of all subject. Though sharing the image data on a public repository such as XNAT is usually desirable, it may not be needed for a small pilot study such as this one.
**Please see above, we have uploaded the dataset file on** [**https://data.mendeley.com/**](https://data.mendeley.com/)**, with the appropriate original data file relevant to this study (DOI:10.17632/2bvdcspxx2.1); it can be accessed via** <https://data.mendeley.com/datasets/2bvdcspxx2/1>

2. Could the authors explicitly specify how the triplanar measurement was performed on MRI? (presumably according to PI-RADS 2.1 recommendations?) This has a small influence on the volume estimate (see Ghafoor et al., 10.1016/j.acra.2020.07.027)
**This was following the PI-RADS version 2.1 guidelines ellipsoid formulation. We have clarified this in the methodology as below, and used the relevant PI-RADS v2.1 reference.
*“…following the PI-RADS version 2.1 guidelines ellipsoid formulation; calculated using (maximum AP dimension) x (maximum longitudinal dimension) [both placed on the mid-sagittal T2W image] x (maximum transverse dimension) [placed on the axial T2W image] x 0.52 [Ref].”*
We have also mentioned the potential for differences between v2.0 and v2.1 in the limitations section and referenced the suggested paper.**

3. The authors could add Bland-Altman plots, which would make it easy for the reader to visually grasp the disagreement between the methods (residual error may be heteroscedastic)
**We have now performed a Bland-Altman Agreement, detailed in the methods and results with representative supplemental figures uploaded. Added:
Methods:
*“Bland-Altman Agreement (BAA) was also used to measure how closely the US derived prostate volumes (TA, TP, TA/TP and TP-TA) compared to the gold-standard MRI-derived prostate volume.”*
Results:
*“Good agreement was demonstrated between all US derived prostate volumes and gold-standard MRI-derived volumes calculated using whole gland segmentation software. A maximum of 3/64 cases lay outside the 95% confidence interval (CI) of agreement lines, with the highest agreement demonstrated between MRI and US-derived prostate volumes using either combined TA-TP or transperineal alone volumes (Supplemental Figure 1). Good inter-reader agreement was shown for readers’ MRI and US-derived prostate volumes, with the greatest agreement noted between US-TP and US TP-TA volumes. Poorest agreement was demonstrated for the US-TA volumes (Supplemental Figure 2).”***

4. If the authors see any additional value in it, they could either just discuss or even perform post hoc estimation of the TZ volume and TZPSAD, which may be slightly superior to whole gland PSAD (see Schneider et al, 10.1016/j.ejrad.2019.108660).
**While TZ volume may better show better correlation, both whole gland (WG) volume and TZ volume show a similar increase with age (Bura, et al Eur Radiol. 2021;31(7):4908-4917), allowing for the confounding factor of an enlarged TZ variably causing compression of the PZ to differing degrees between patients. Furthermore, the TZ cannot be reliably assessed with Ultrasound (especially retrospectively in this cohort), and WG derived PSA-density is currently more widely used in clinical practice. We have added these references and paraphrased this discussion within the limitations.**

5. Re "limiting the accuracy of the segmentation-derived volume" [Results; p14]: This is rather an expression of the limited functionality of the software used than a true limitation of MRI. TURP defects are well delineated on T2w imaging. With state-of-the-art software such as ImageJ, accurate segmentations can still be provided.
**The MRI volumetric measurements were considered accurate and rather it was the US measurements that were problematic, however, the reviewer’s comment is noted and we have added this to the discussion section.**

6. PLOS authors have the option to publish the peer review history of their article ([what does this mean?](https://journals.plos.org/plosone/s/editorial-and-peer-review-process#loc-peer-review-history)). If published, this will include your full peer review and any attached files.


If you choose “no”, your identity will remain anonymous but your review may still be made public.


**Do you want your identity to be public for this peer review?** For information about this choice, including consent withdrawal, please see our [Privacy Policy](https://www.plos.org/privacy-policy).

Reviewer #1: **Yes:** Anton S. Becker, MD, PhD

[NOTE: If reviewer comments were submitted as an attachment file, they will be attached to this email and accessible via the submission site. Please log into your account, locate the manuscript record, and check for the action link "View Attachments". If this link does not appear, there are no attachment files.]

While revising your submission, please upload your figure files to the Preflight Analysis and Conversion Engine (PACE) digital diagnostic tool, <https://pacev2.apexcovantage.com/>. PACE helps ensure that figures meet PLOS requirements. To use PACE, you must first register as a user. Registration is free. Then, login and navigate to the UPLOAD tab, where you will find detailed instructions on how to use the tool. If you encounter any issues or have any questions when using PACE, please email PLOS at [figures@plos.org](mailto:figures@plos.org). Please note that Supporting Information files do not need this step.
